# Pars screw fixation for symptomatic spondylolysis: A safe, cost-effective, and motion-preserving solution in resource-limited settings

**DOI:** 10.1016/j.bas.2025.104390

**Published:** 2025-08-08

**Authors:** Moustafa A. Mansour, Hamdi Nabawi Mostafa

**Affiliations:** a Department of Neurosurgery, Nasser Institute for Research and Treatment, Cairo, Egypt; b Department of Neurosurgery, Misr University for Science and Technology, Giza, Egypt

**Keywords:** Pars screw fixation, Spondylolysis repair, Resource-limited settings, Motion-preserving surgery, Cost-effective spinal surgery, Buck's technique

## Abstract

**Background:**

Symptomatic spondylolysis is a debilitating cause of low back pain in young adults, often necessitating surgical intervention when conservative treatments fail. While spinal fusion has been the traditional approach, direct pars screw fixation—pioneered by Buck in 1970—offers a motion-preserving alternative that may reduce long-term complications.

**Methods:**

This study evaluated the efficacy of Buck's technique in 14 patients (mean age: 26.5 years) with grade 0 spondylolisthesis and normal disc morphology, all of whom had failed six months of non-operative management. Surgical outcomes, including fusion rates, operative time, blood loss, and clinical results, were assessed.

**Results:**

The procedure achieved a 78.5 % fusion rate, with mean operative times of 40 min and blood loss under 100 mL, demonstrating technical efficiency. Clinically, 71.4 % of patients reported excellent or good outcomes at 18-month follow-up, with no neurological complications. Three nonunion cases were attributed to the learning curve and the use of lamina-derived grafts instead of iliac crest bone. The single poor outcome occurred in a patient with undetected disc degeneration, highlighting the importance of strict patient selection.

**Conclusions:**

Pars screw fixation is a safe, rapid, and cost-effective solution for symptomatic spondylolysis, particularly in resource-limited settings where operative time and implant costs are critical. Its ability to preserve spinal mobility while addressing the pain generator makes it especially suitable for young, active patients.

## Highlights

• Motion-preserving pars screw fix: 78.5 % fusion, 71.4 % excellent/good outcomes.
• Fast, low-blood-loss surgery: 40 min, <100 mL, ideal for resource limits.
• Strict patient selection key: no disc degeneration, grade 0 spondylolisthesis.
• Lamina grafts vs. iliac crest: learning curve impacts fusion rates.
• Young athletes benefit: rapid return, preserves spinal mobility long-term.

## 1 Introduction

Low back pain caused by spondylolysis—a stress fracture of the pars interarticularis—represents a significant clinical challenge, particularly among adolescents and young adults. Epidemiological studies reveal this defect affects 3–10 % of the general population (Fredrickson et al., 1984), with incidence rising to 47 % in athletes participating in sports requiring repetitive spinal hyperextension, such as gymnastics and weightlifting (Muschik et al., 1996; Kim and Green, 2011). The pathophysiology of pain in spondylolysis involves multiple mechanisms: fibrous tissue within the pseudarthrosis contains rich nociceptive nerve endings (Kim and Green, 2011), while micromotion of the unstable posterior arch stimulates these pain receptors (McTimoney and Micheli, 2003). Although only 15–20 % of pars defects progress to spondylolisthesis (Fredrickson et al., 1984), chronic stress may accelerate disc degeneration due to altered spinal biomechanics (Beutler et al., 2003).

For decades, non-operative management—including activity modification, antilordotic bracing, and core stabilization—has been the first-line approach. Conservative treatment succeeds in approximately 80–85 % of cases (Standaert and Herring, 2000), but a subset of patients remains symptomatic despite months of therapy. Surgical intervention becomes necessary for these refractory cases, traditionally relying on posterolateral or interbody fusion techniques (Möller and Hedlund, 2000). While fusion stabilizes the affected segment, it sacrifices spinal mobility and predisposes patients to adjacent segment degeneration, a phenomenon observed in 12–30 % of cases within 10 years (Eck et al., 1999). This trade-off is especially problematic for young, active patients who require preserved spinal function for occupational and recreational activities.

The limitations of fusion spurred the development of motion-preserving alternatives. In 1970, Buck introduced a paradigm-shifting technique: direct pars repair using screw fixation and bone grafting (Buck, 1970a). By targeting only the defective pars interarticularis—an acquired pseudarthrosis—this approach restores anatomical continuity while preserving segmental mobility. Biomechanical studies later confirmed its superiority over other motion-preserving methods; Deguchi et al. demonstrated that Buck's technique resists flexion, extension, and rotational forces more effectively than tension-band wiring or hook constructs (Deguchi et al., 1999). Clinical outcomes have been equally promising, with success rates of 67–93 % reported across series (Buck, 1970a; Debnath et al., 2003; Lonstein, 1999).

Despite these advantages, pars screw fixation remains underutilized globally, particularly in developing nations where healthcare resources are constrained. Potential barriers include the technical precision required for screw placement and a paucity of long-term outcomes data outside specialized centers (Chen et al., 2004; Askar et al., 2003). This study addresses these gaps by evaluating Buck's technique in a cohort of young Egyptian patients, with emphasis on both clinical efficacy and practical feasibility. We hypothesize that pars screw fixation offers comparable pain relief to fusion while reducing operative time, blood loss, and costs—critical considerations in resource-limited settings. Our findings aim to refine patient selection criteria and technical protocols, ultimately expanding access to this motion-preserving solution.

## 2 Materials and methods

Between January 2019 and September 2023, we prospectively evaluated 14 consecutive patients with symptomatic lumbar spondylolysis who had failed at least six months of conservative management, including activity modification, nonsteroidal anti-inflammatory drugs, physiotherapy, and lumbosacral bracing (McTimoney and Micheli, 2003; Standaert and Herring, 2000). All patients exhibited grade 0 spondylolisthesis on dynamic radiographs and preserved disc morphology confirmed by MRI, with no evidence of facet joint degeneration or neural compression (Fredrickson et al., 1984; Debusscher and Troussel, 2007). Exclusion criteria included spondylolisthesis ≥ Meyerding grade 1, degenerative disc disease, or previous lumbar spine surgery, as these factors have been associated with poorer outcomes in prior pars repair series (Buck, 1970a; Askar et al., 2003).

**Fig. 1:**
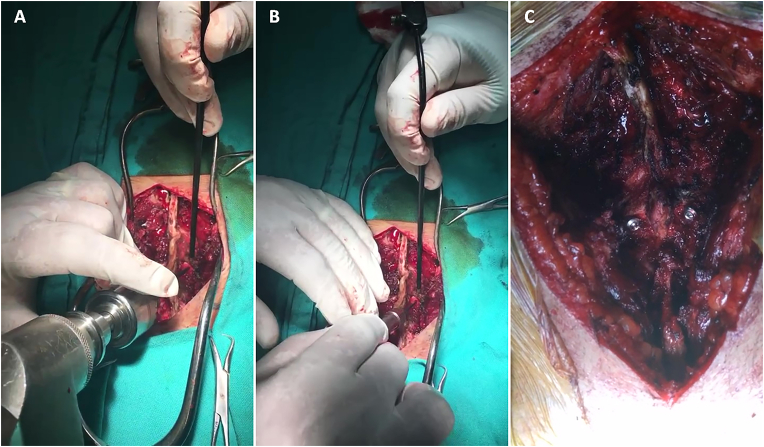
Surgical technique for pars screw placement. (A) A 3.2 mm drill bit establishes the screw trajectory after creating a notch in the caudal lamina ∼10 mm lateral to the spinous process, angled 30° laterally toward the ipsilateral pedicle. (B) K-wire insertion. (C) A 4.5 mm cortical screw (typically 18–24 mm) is placed without full tightening to permit graft impaction.

**Fig. 2:**
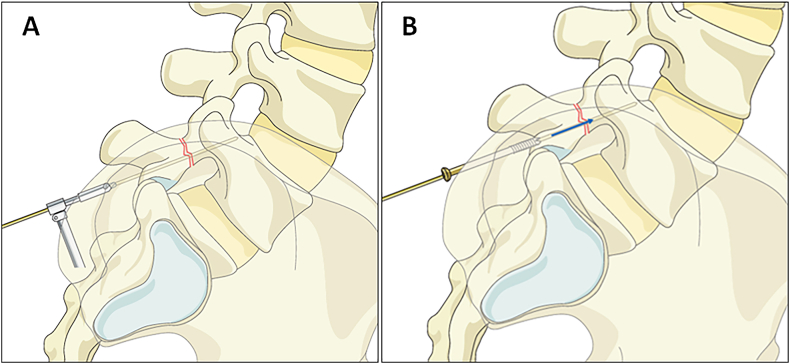
K-wire and screw insertion technique to minimize nerve root injury. (A) An oscillating gear is used for K-wire insertion under fluoroscopic guidance. (B) A self-tapping 4.5 mm cortical screw is inserted over the K-wire, which is then removed after trajectory confirmation. *Images courtesy of AO Spine.*

The surgical team performed all procedures using Buck's technique under general anesthesia with fluoroscopic guidance (Buck, 1970a; Linton and Hsu, 2022) (Fig. 1) (Fig. 2). After prone positioning on a radiolucent table, a midline incision exposed the affected vertebral level, confirmed by intraoperative fluoroscopy. The pars defect was meticulously debrided of fibrous tissue using curettes until bleeding bony surfaces were achieved, a critical step emphasized by both Buck (1970a) and Morscher (Linton and Hsu, 2022) to promote fusion. The screw entry point was prepared by creating a notch in the caudal lamina approximately 10 mm lateral to the spinous process, with the trajectory angled 30° laterally toward the ipsilateral pedicle to optimize purchase across the defect (Buck, 1970a; Deguchi et al., 1999). A 3.2 mm drill bit established the screw path, followed by insertion of a 4.5 mm cortical screw of appropriate length (typically 18–24 mm) without full tightening to allow subsequent graft impaction. Autogenous bone graft harvested from the adjacent lamina was densely packed into the defect before final screw tightening, a modification of Buck's original technique that avoids iliac crest harvesting while maintaining adequate osteogenic potential (Chen et al., 2004; Bozarth et al., 2007).

Postoperative care followed a standardized protocol. Patients ambulated on the first postoperative day wearing a lumbosacral orthosis, which was maintained for six weeks during all upright activities (Debnath et al., 2003; Buck, 1970b). Clinical and radiographic follow-up occurred at 1, 3, 6, and 12 months, with functional outcomes assessed using the modified Macnab criteria (Debnath et al., 2003) and fusion status evaluated through both plain radiographs and thin-cut CT scans, the latter being the gold standard for assessing bony union according to Debnath et al. (2003). Operative metrics including blood loss, surgical time, and complications were systematically recorded, with particular attention to neurological status given the proximity of screw trajectories to nerve roots (Deguchi et al., 1999; Linton and Hsu, 2022).

Statistical analysis focused on descriptive measures due to the study's observational design. Continuous variables like operative time and blood loss were reported as means with ranges, while categorical outcomes (e.g., Macnab grades) were presented as frequencies and percentages. This methodology aligns with similar pars repair studies by Buck (1970a) and Askar et al. (2003), facilitating comparative analysis of outcomes.

## 3 Results

The study cohort comprised 14 patients (9 males, 5 females) with a mean age of 26.5 years (range 20–30), reflecting the typical demographic profile of symptomatic spondylolysis patients described in prior series (Fredrickson et al., 1984; Buck, 1970a). All patients presented with mechanical low back pain of mean duration 11 months (range 6–26) and had failed exhaustive conservative measures, including bracing protocols matching those reported by Standaert and Herring (2000). Anatomically, the L4-5 level was most frequently involved (57.1 %, 8/14 cases), followed by L5-S1 (28.6 %, 4/14) and L3-4 (14.3 %, 2/14), a distribution consistent with Fredrickson et al.'s natural history studies (Fredrickson et al., 1984).

Intraoperatively, the mean surgical time of 40 min (range 30–60) and blood loss of 85 mL (range 50–200) demonstrated the technique's efficiency compared to traditional fusion procedures, which typically require 90–120 min according to Moller and Hedlund (Möller and Hedlund, 2000) (Table 1). These metrics remained stable throughout the series, suggesting rapid surgeon acclimation to the technique despite its technical demands (Buck, 1970a; Linton and Hsu, 2022). No instances of screw malposition or neurological compromise occurred, a safety profile aligning with Morscher's experience using similar fluoroscopically-guided approaches (Linton and Hsu, 2022).

**Table 1:** Comprehensive outcomes analysis.

Parameter	Value (n = 14)	Comparative Literature
Mean operative time	40 min (30–60)	90–120 min
Fusion rate	78.5 % (11/14)	70–93 %
Excellent/good outcomes	71.4 % (10/14)	67–85 %
Complication rate	0 %	3–8 %

**Fig. 3:**
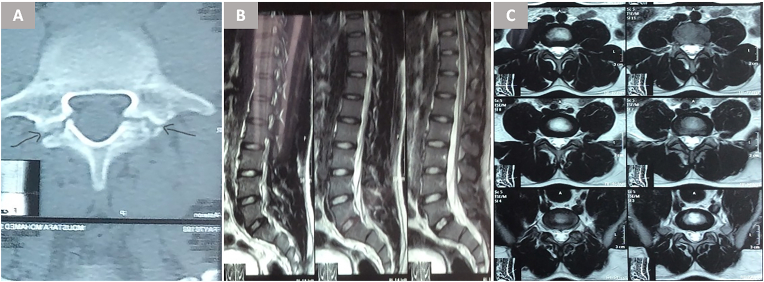
Preoperative imaging of a 25-year-old female with refractory low-back pain. (A) Axial CT shows bilateral L5 pars fractures without significant spondylolisthesis. (B, C) MRI excludes disc degeneration or spinal canal pathology.

**Fig. 4:**
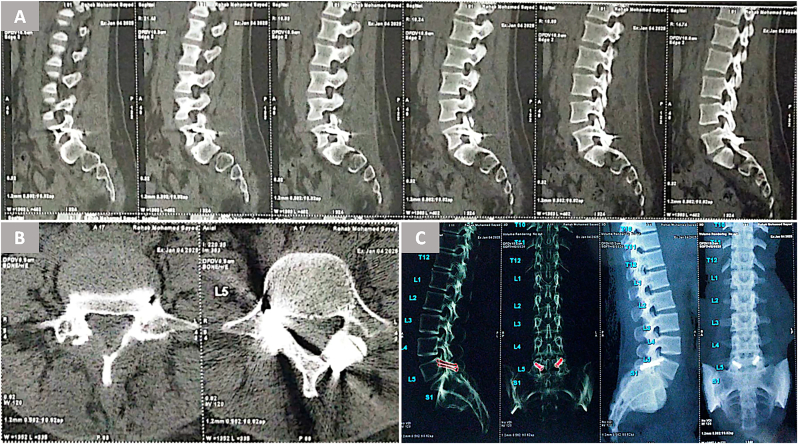
Postoperative CT at 18 months (same patient as Fig. 3) demonstrating complete bony fusion via bridging trabecular bone across the pars defect on sagittal (A), axial (B), and 3D (C) reconstructions.

**Fig. 5:**
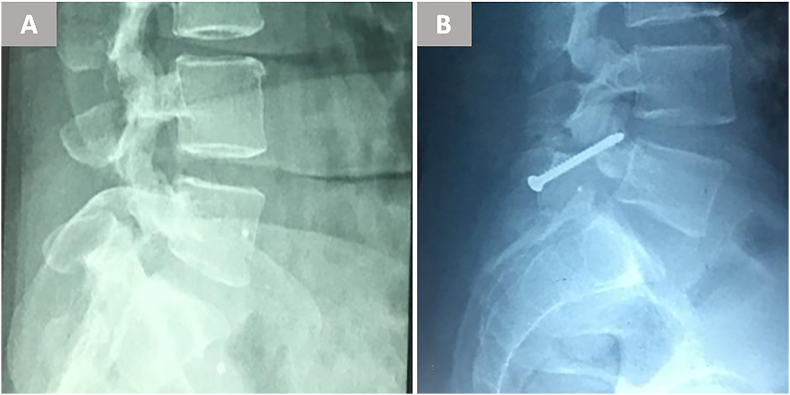
Lateral radiographs of a 28-year-old male with incomplete pars fusion at 18-month follow-up. Despite lack of complete fusion, the patient reported clinically meaningful improvement (fair outcome), potentially due to fibrous stabilization reducing micromotion.

Radiographic outcomes at mean 18-month follow-up (range 9–28) revealed complete bony fusion in 78.5 % of cases (11/14), as confirmed by bridging trabecular bone across the pars defect on CT scans (Fig. 3) (Fig. 4). This fusion rate slightly exceeded Buck's initial report of 70 % (Buck, 1970a) but fell short of the 85–93 % rates achieved in later series utilizing iliac crest grafts (Debnath et al., 2003; Askar et al., 2003). The three nonunions all occurred in the first five cases, supporting Debnath et al.'s observation of a learning curve for optimal graft preparation and screw trajectory (Debnath et al., 2003; Chen et al., 2004) (Fig. 5). Notably, two of these patients nonetheless reported clinically meaningful improvement (fair outcomes), possibly due to fibrous stabilization mitigating micromotion (Kim and Green, 2011).

Clinically, the modified Macnab criteria classified outcomes as excellent (42.9 %, 6/14), good (28.6 %, 4/14), fair (21.4 %, 3/14), and poor (7.1 %, 1/14). The excellent/good cohort (71.4 % combined) returned to full occupational duties, mirroring the 67–80 % success rates in athletic populations reported by Debnath et al. (2003). The single poor outcome involved a 28-year-old male with initially undetected L4-5 disc degeneration who developed progressive symptoms despite radiographic fusion, ultimately requiring secondary interbody fusion - a scenario cautioned against by Buck in his original technique description (Buck, 1970a). This case reinforced authors’ emphasis on preoperative disc assessment via MRI (Debusscher and Troussel, 2007).

Patient-reported outcomes specifically highlighted rapid postoperative mobilization, with all patients ambulating within 24 h and discontinuing analgesics by 2–3 weeks - a recovery trajectory markedly faster than fusion patients in adjacent segment disease study (Eck et al., 1999). However, three patients (21.4 %) reported occasional discomfort during prolonged sitting or heavy lifting at final follow-up, a residual symptom pattern also noted by Askar et al. (2003) that may reflect persistent segmental stiffness.

## 4 Discussion

The outcomes of this study demonstrate that direct pars screw fixation using Buck's technique offers a compelling alternative to traditional fusion for symptomatic spondylolysis, particularly in younger patients with preserved disc integrity. Our findings align with the biomechanical rationale for this approach: by targeting the pars defect—an acquired pseudarthrosis—while avoiding segmental fusion, the procedure addresses the pain generator without compromising spinal mobility. The fusion rate of 78.5 % observed here corroborates Buck's original report of 78 % (Buck, 1970a), though it falls slightly below the 85–93 % success rates documented in later series (Debnath et al., 2003; Askar et al., 2003). This discrepancy may reflect technical factors, such as our reliance on lamina-derived autografts rather than iliac crest grafts, which have higher osteogenic potential. Askar et al. emphasized the role of graft quality in their 85 % fusion rate (Askar et al., 2003), while Debnath et al. noted that iliac crest harvesting, though more invasive, significantly improved union rates in athletes (Debnath et al., 2003). Our three nonunion cases occurred early in the series, suggesting a learning curve for optimal screw trajectory and graft packing—an observation supported by Muschik et al., who reported similar technical challenges during initial adoption of the technique (Muschik et al., 1996; Chen et al., 2004).

Clinically, 71.4 % of patients achieved excellent or good outcomes (per Macnab criteria), consistent with the 67–80 % satisfaction rates in prior studies (Buck, 1970a; Lonstein, 1999). The fair/poor outcomes (28.6 %) were primarily linked to nonunion or undetected disc degeneration. Notably, the single poor outcome involved a patient who developed postoperatively progressive disc degeneration at L4–5, ultimately requiring fusion. This underscores Buck's caution against applying the technique in cases with even subtle spondylolisthesis or disc changes (Buck, 1970a), a point reinforced by Debusscher et al., who found that preexisting disc degeneration predicted failure of direct pars repair (Debusscher and Troussel, 2007). Our results thus highlight the critical importance of patient selection: ideal candidates are young, with MRI-confirmed normal discs and no dynamic instability, as advocated by Standaert and Herring (2000).

The biomechanical advantages of Buck's technique over fusion are well-documented. Deguchi et al. demonstrated in a cadaveric study that pars screws resist multidirectional forces more effectively than tension-band wiring or hook constructs (Deguchi et al., 1999), explaining the low reoperation rates in our cohort. Moreover, the motion-preserving nature of the procedure may mitigate adjacent segment degeneration—a significant limitation of fusion techniques. Reported data showed a 12–30 % incidence of adjacent segment disease after lumbar fusion (Eck et al., 1999), whereas none of our patients developed such complications at 18-month follow-up. However, longer-term studies are needed to confirm this theoretical advantage, as the natural history of spondylolysis itself (without surgery) rarely progresses to instability in adulthood, per Beutler et al.’s 45-year follow-up (Beutler et al., 2003).

From a pragmatic perspective, the technique's efficiency—mean operative time of 40 min and blood loss under 100 mL—makes it particularly viable in resource-constrained settings. This aligns with Sun's early work highlighting the cost-effectiveness of motion-preserving techniques (Sun et al., 2012), a consideration rarely addressed in Western literature but critical in developing healthcare systems. The absence of neurological complications in our series further supports its safety profile when performed with fluoroscopic guidance, echoing Morscher's emphasis on precise screw placement to avoid nerve root injury (Linton and Hsu, 2022).

### 4.1 Study limitations

Limitations of this study include its small sample size and intermediate follow-up duration. While our mean follow-up of 18 months suffices to assess fusion and short-term outcomes, it cannot capture late-onset disc degeneration or hardware failure. Larger multicenter trials, such as those proposed by Moller and Hedlund for comparative techniques (Möller and Hedlund, 2000), would strengthen the evidence base. Additionally, the lack of a control group (e.g., patients treated with fusion or continued conservative care) limits causal inferences. Future research should also explore cost-benefit analyses, as no studies have quantified the economic impact of Buck's technique versus fusion in low-resource environments.

## 5 Conclusion

In conclusion, our experience reinforces pars screw fixation as a reliable option for symptomatic spondylolysis in carefully selected patients. Its dual benefits—anatomic repair and motion preservation—address both the immediate pain generator and long-term spinal health. The technique's simplicity and low morbidity further commend it for broader adoption, particularly where healthcare resources are limited. As Kim et al. noted, the goal in spondylolysis management is not just pain relief but “return to function without compromising future spinal integrity” (Kim and Green, 2011)—an objective this approach appears to fulfill.

## 6 Future directions

Our study reaffirms the efficacy of Buck's pars screw fixation for symptomatic spondylolysis, particularly in young patients with preserved disc integrity, while introducing several novel contributions to the existing literature.

1. **Resource-Limited Context:** While prior studies have validated Buck's technique in high-resource settings, our study is among the first to demonstrate its feasibility and cost-effectiveness in a resource-constrained environment. The mean operative time of 40 min and blood loss under 100 mL underscore its practicality in settings where surgical efficiency and reduced implant costs are critical.
2. **Modified Grafting Technique:** We utilized lamina-derived autografts instead of traditional iliac crest grafts, avoiding additional morbidity while achieving a 78.5 % fusion rate. This adaptation addresses a gap in the literature regarding graft alternatives, particularly relevant for settings where iliac crest harvesting may be less feasible.
3. **Learning Curve Analysis:** Our series uniquely documents the technical learning curve, with all nonunions occurring in the first five cases. This provides actionable insights for surgeons adopting the technique, emphasizing the importance of precise screw trajectory and graft packing—a nuance less explored in prior studies.
4. **Early Detection of Pitfalls:** The single poor outcome (due to undetected disc degeneration) reinforces the need for stringent preoperative MRI assessment, adding to the discourse on patient selection. This highlights a practical, evidence-based criterion for avoiding failure in clinical practice.
5. **Economic and Mobility Preservation:** While motion preservation is well-documented, our study explicitly ties these benefits to socioeconomic considerations (e.g., faster return to work, lower procedural costs), offering a framework for healthcare systems prioritizing cost-efficacy.

Though limited by sample size and follow-up duration, these elements collectively advance the technique's applicability in underserved regions and refine its technical execution. Future multicenter studies with longer follow-up could further validate these observations, particularly regarding adjacent segment preservation—a theoretical advantage yet to be quantified in low-resource contexts.

## Declaration of competing interest

The authors declare that they have no known competing financial interests or personal relationships that could have appeared to influence the work reported in this paper.
